# CLINICAL AND SURGICAL DILEMMAS IN OCTOGENARIAN PATIENTS WITH SMALL BOWEL OBSTRUCTION

**DOI:** 10.1590/0102-672020240008e1801

**Published:** 2024-05-20

**Authors:** Tomás GONZÁLEZ-ARESTIZÁBAL, Álvaro MORALES, Tania AVAYÚ-ZALIASNIK, Attila CSENDES, Owen KORN, Manuel FIGUEROA-GIRALT

**Affiliations:** 1 Universidad de Chile, Clinical Hospital, Department of Surgery, Santiago, Chile.

**Keywords:** Intestinal Obstruction, Octogenarians, Mortality, Health Care Costs, Obstrução Intestinal, Octogenários, Mortalidade, Custos de Cuidados de Saúde

## Abstract

**BACKGROUND:**

Small bowel obstruction (SBO) is a major problem in emergencies. Comorbidities increase morbimortality, which is reflected in higher costs. There is a lack of Latin American evidence comparing the differences in postoperative results and costs associated with SBO management.

**AIMS:**

To compare the risk of surgical morbimortality and costs of SBO surgery treatment in patients older and younger than 80 years.

**METHODS:**

Retrospective analysis of patients diagnosed with SBO at the University of Chile Clinic Hospital from January 2014 to December 2017. Patients with any medical treatment were excluded. Parametric statistics were used (a 5% error was considered statistically significant, with a 95% confidence interval).

**RESULTS:**

A total of 218 patients were included, of which 18.8% aged 80 years and older. There were no differences in comorbidities between octogenarians and non-octogenarians. The most frequent etiologies were adhesions, hernias, and tumors. In octogenarian patients, there were significantly more complications (46.3 vs. 24.3%, p=0.007, p<0.050). There were no statistically significant differences in terms of surgical complications: 9.6% in <80 years and 14.6% in octogenarians (p=0.390, p>0.050). In medical complications, a statistically significant difference was evidenced with 22.5% in <80 years vs 39.0% in octogenarians (p=0.040, p<0.050). There were 20 reoperated patients: 30% octogenarians and 70% non-octogenarians without statistically significant differences (p=0.220, p>0.050). Regarding hospital stay, the average was significantly higher in octogenarians (17.4 vs. 11.0 days; p=0.005, p<0.050), and so were the costs, being USD 9,555 vs. USD 4,214 (p=0.013, p<0.050).

**CONCLUSIONS:**

Patients aged 80 years and older with surgical SBO treatment have a higher risk of medical complications, length of hospital stay, and associated costs compared to those younger.

## RESUMO

**RACIONAL:**

A obstrução do intestino delgado (OID) é um grande problema em emergências. As comorbidades aumentam a morbimortalidade, o que se reflete no aumento dos custos. Há uma falta de evidência latino-americana comparando as diferenças nos resultados pós-operatórios e custos associados ao manejo da OID.

**OBJETIVOS:**

Comparar o risco de morbimortalidade cirúrgica e os custos de tratamento cirúrgico da OID em pacientes com idade inferior e superior a 80 anos.

**MÉTODOS:**

Análise retrospectiva de pacientes diagnosticados com OID no Hospital Clínico da Universidade do Chile entre janeiro de 2014 e dezembro de 2017. Pacientes com qualquer tratamento médico foram excluídos. Estatísticas paramétricas foram usadas: erro de 5% foi considerado estatisticamente significante, com intervalo de confiança de 95%.

**RESULTADOS:**

Um total de 218 pacientes foram incluídos, dos quais 18,8% tinham 80 anos ou mais. Não houve diferenças nas comorbidades entre octogenários e não octogenários. As etiologias mais frequentes foram aderências, hérnias e tumores. Nos pacientes octogenários houve significativamente mais complicações (46,3 vs. 24,3%, p=0,007, p<0,050). Não houve diferenças estatisticamente significativas quanto às complicações cirúrgicas: 9,6% em <80 anos e 14,6% em octogenários (p=0,390, p>0,050). Nas complicações médicas foi evidenciada diferença estatisticamente significativa com 22,5% em <80 anos vs. 39.0% em octogenários (p=0,040, p<0,050). Foram reoperados 20 pacientes: 30% octogenários e 70% não octogenários, sem diferenças estatisticamente significativas (p=0,220, p>0,050). Em relação ao tempo de internação, a média foi significativamente maior nos octogenários (17,4 v/s 11,0 dias; p=0,005, p<0,050). Da mesma forma, também foram os custos com 9.555 USD vs. 4.214 USD (p=0,013, p<0,050).

**CONCLUSÕES:**

Pacientes com 80 anos ou mais com tratamento cirúrgico de OID têm maior risco de complicações médicas, tempo de internação e custos associados em comparação aos mais jovens.

## INTRODUCTION

Small bowel obstruction (SBO) is a major problem in surgical emergencies, representing about 20% of all causes of surgical acute abdomen^1^. The most frequent etiologies are adhesions, hernias (abdominal wall and internal), and tumors. The diagnosis is based on considering risk factors, symptoms, physical examination, and laboratory tests, in addition to computed tomography (CT) with contrast to evaluate the etiology and assess the presence of possible ischemia^3,9^, determining the need or not for surgical treatment^3,9^.

Currently, the population is aging progressively worldwide. There were 703 million people aged 65 years and over in 2019. It is estimated that the number of elderly people will more than double, reaching more than 1.5 billion by 2050. At the national level, according to the 2017 Census survey, there were 2,003,256 older adults (≥65 years old), that is, 11.4% of the total country population. By 2035, a significant increase in this age group is expected, forecasting figures reaching 3,993,821 older adults, which would be equivalent to 18.9% of the total population^7^.

Another element to consider is that the comorbidities associated with these patients not only complicate the care and treatment of surgical pathology but also increase the risk of surgical morbidity and mortality^4^. This is reflected in the increase in costs associated with the care of surgical pathology, which can reach 2–3 billion dollars in total monetary costs in the United States. There are few reports in Latin America about this topic. In Chile, there are no published studies that compare postoperative outcomes and costs in SBO between octogenarian and non-octogenarian groups. However, a previous comparative study from our group showed greater associated complications and costs in octogenarian patients^6^.

The primary objective of this study was to compare the risk of surgical morbidity and mortality in patients younger than and aged 80 years and older with a diagnosis of SBO treated surgically. The secondary aim was to compare the costs associated with health care in both age groups.

## METHODS

Retrospective analysis of clinical records of patients diagnosed with SBO at the University of Chile Clinical Hospital from January 2014 to December 2017. Hospitalized patients older than 15 years with a diagnosis of SBO of all etiologies who received surgical treatment were included. They were divided into two groups: octogenarians (at least 80 years old) and non-octogenarians (<80 years old).

The exclusion criteria were patients with gastric obstructions, lower intestinal obstructions, those who received only medical treatment, and patients with incomplete clinical record information. The definitions used in this study are the same as those used by our research group in a previous publication^6^.

Descriptive and analytical statistics were used. Demographic variables of the sample such as age, sex, and comorbidities were described. Outcomes analyzed were length of stay, etiology, hospitalization costs (equivalence of Chilean pesos to American dollars at submission day), mortality, postoperative adverse events, and number of reoperations.

For comparisons between both groups, the chi-square test (χ^2^), Student’s t-test, and Shapiro-Wilk test were used as appropriate. A 5% error was considered statistically significant, with a 95% confidence interval. The analysis and graphs were performed with Graph-Pad Prism^®^. Considering the retrospective nature of the study, privacy, and the anonymous analysis of all records, there was no need for Institutional Review Board approval.

## RESULTS

The present study included a total of 218 patients, of which 41 (18.8%) were octogenarians and 177 (81.2%) non-octogenarians. Table 1 shows the comparative differences in comorbidities with no statistically significant differences between the two groups.

**Table 1:** Patients’ description.

Variable	<80 years n (%)	≥80 years n (%)	p-value	OR	95%CI
Total	177 (81.2)	41 (18.8)			
Male	72 (40.6)	11 (26.8)	0.09	1.21	0.727–2.148
Female	105 (89.7)	30 (73.1)	0.09		
Hypertension	60 (34)	20 (49)	0.85	0.93	0.447–1.946
Diabetes mellitus	27 (15)	4 (10)	0.82	0.60	0.216–1.774
Obesity	34 (19)	5 (12)	0.29	0.58	0.216–1.774
Smoking	29 (16)	2 (5)	0.05	0.26	0.059–1.027

OR: odds ratio.

Regarding the etiologies of intestinal obstruction for both age groups, it was found that adhesions were the most frequent, followed by hernias and, lastly, by tumors. When comparing these etiologies according to age, hernias comprised a higher percentage in octogenarian patients (p=0.020; p<0.050) (Table 2).

**Table 2:** Etiologies.

Etiology	<80 years n (%)	≥80 years n (%)	p-value	OR	95%CI
Adherences	103 (58)	22 (54)	0.59	0.83	0.426–1.666
Hernia	22 (12)	11 (27)	0.02	2.58	1.188–5.961
Tumor	22 (12)	4 (10)	0.63	0.76	0.270–2.350

OR: odds ratio.

There were no significant differences regarding intestinal resection need (octogenarian group 34.1 vs 27.1% in non-octogenarians; p=0.580; p>0.050).

The general analysis of postoperative adverse events (POAE) showed that octogenarian patients presented a higher percentage of global complications (46.3 vs 24.2%; p=0.0048, p<0.050). In the subgroup analysis, there was also a higher percentage of medical POAE in octogenarians than in the younger age group (22.5 vs 39.0%; p=0.045, p<0.050) (Table 3).

**Table 3:** Complications.

Variable	<80 years	≥80 years	p-value	OR	95%CI
Global complications	43/177 (24.3%) (24.2%)	19/41 (46.3%)	0.007	2.69	1.292–5.489
Surgical POAE	17 (9.6%)	6 (14.6%)	0.395	1.61	0.6001–4.480
Clavien Dindo Surgical	I	I			
	II	II			
	III (14)	III (6)			
	IV (2)	IV			
	V (1)	V			
Medical POAE	40 (22.5%)	16 (39.0%)	0.045	2.19	1.069–4.522
Clavien-Dindo Medical	I (3)	I (2)			
	II (18)	II (6)			
	III (10)	III (3)			
	IV (7)	IV (3)			
	V (2)	V (2)			
Clavien-Dindo I, II	21 (52.5%)	8 (50%)			
Clavien-Dindo III–V	19 (47.5%)	8 (50%)			

POAE: postoperative adverse events; OR: odds ratio.

The general causes of reoperation were mostly due to anastomosis leakage (9.7% octogenarians vs 2.8% non-octogenarians) without significant differences (p=0.160; p>0.050), followed by intestinal perforation 2.4 vs 1.1%, respectively (p=0.900; p>0.050). Although there was evidence of a greater tendency for reoperation in octogenarians (14.6 vs 11.9%), this difference was not significant (p=0.690; p>0.050).

Despite mortality being higher in older patients, this difference was also not significant (4.2 vs 1.8%; p=0.660, p>0.050).

**Figure 1:**
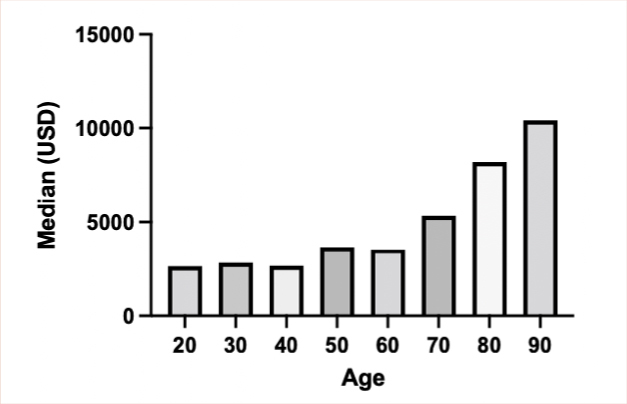
Hospitalization costs (United States dollar) by age decades.

It was shown that the average number of days of hospitalization was significantly higher in octogenarians with an average of 17.4 days vs. 11.0 days (p=0.005; p<0.050) (Table 4). Total hospitalization costs were different when compared by age. The median of the octogenarians was USD 8,189.57 with a range of USD 721.41–70,390.36 and of non-octogenarians was USD 3,609.03 with a range of USD 1,375.81–98,837.48 (p=0.013, p<0.050), corresponding to 2.27 times more expensive (Table 5). When stratified by decades, a direct relationship between older age and hospitalization costs was obtained (Figure 1).

**Table 4:** Hospitalization length and total costs.

Variable	<80 years	≥80 years	p-value
Days of hospitalization	11 (1–115)	17.4 (2–103)	0.0005
Total costs USD: Median (range)	3,609.03 (1,375.81–98,837.48)	8,182.57 (721.41–70,390.36)	0.013
Surgical complication costs USD	17,067.28 (2,588.28–98,837.48)	32,432.64 (18,143.49–46,721.79)	0.580
Medical complication costs USD	22,104.65 (4,330.79–98,837.48)	13,499.81 (4,880.62–70,930.36)	0.960

USD: United States dollar.

**Table 5:** Mean, median, and interquartile range of costs (United States dollar) stratified by decade.

	20–29	30–39	40–49	50–59	60–69	70–79	80–89	90–99
Mean	2,781.02	4,629.53	6,470.78	8,033.72	6,207.39	14,175.92	16,258.04	12,786.68
Median	2,643.36	2,835.13	2,668.33	3,683.99	3,548.61	5,330.90	8,182.57	10,408.34
25% IQ	2,402.51	1,418.09	1,739.99	1,431.64	1,375.81	1,871.68	721.41	2,818.90
75% IQ	3,671.98	27,644.60	39,318.18	44,348.29	51,834.70	98,837.48	70,390.36	29,475.56

IQ: interquartile range.

## DISCUSSION

Intestinal obstruction is a common cause of emergency room visits and emergency operations^13^. Within this group, in this series, around a fifth of the patients were octogenarians. As it is already known, this age group is more prone to comorbidities and frailty. In addition, these patients may present late in the evolution of the disease, with atypical or unspecific symptoms^8^, sometimes leading to late diagnoses and surgical interventions, which is associated with worse results^10^.

An increase in morbidity and mortality has been described concerning emergency surgery in elderly patients, previously described in other reports^2,6,8,11^. An example of this was presented in the article by Figueroa-Giralt et al.^7^ in which risk factors in SBO were evaluated, being over 80 years of age an independent factor for postoperative complications, as well as the C-reactive protein >70 mg/dL.

It is remarkable that in this series, there were no significant differences between the two groups regarding surgical complications and reoperation rate; instead, the burden of medical complications was more relevant in the group of octogenarians, which corroborates international studies^5,11^. One possible explanation is the small sample size. If it had been greater, maybe a statistical significance would have been achieved in terms of anastomotic leaks, reoperation, and mortality.

Regarding costs, it is shown a clear tendency to increase as age increases, marking a direct relationship between older age and a higher final cost of hospitalization. This result is consistent with that described by Csendes Juhasz et al.^2^ where surgical patients >80 years of age had costs 3.7 times higher than the younger group.

Along with this, there is an increase in the days of hospitalization in octogenarian vs non-octogenarian patients. Unlike what was observed by Quero et al., who did not show a difference in hospital stay in patients >80 years of age with acute intestinal obstruction^12^.

Accordingly, it is possible to state that the increase in medical complications in octogenarian patients operated on for intestinal obstruction determines a longer hospital stay and is associated with a higher total cost of hospitalization. Thus, it can be deduced that the direct search to reduce medical complications in octogenarian patients with SBO, who underwent surgical treatment, could significantly reduce hospitalization costs.

There is a need to analyze strategies to reduce medical complications and costs associated with hospitalizations due to surgically resolved intestinal obstruction in octogenarian patients.

The strengths of this study lie in being one of the few international reports and, to the best of the authors’ knowledge, the only Latin American report published that compares age-related complications and costs in patients with SBO surgically treated. The limitations derive from being a retrospective study without long-term follow-up.

## CONCLUSION

Patients 80 years of age and older have a higher risk of medical complications after surgery for upper intestinal obstruction, which entails a longer stay and hospital costs.
